# Choosing an appropriate bacterial typing technique for epidemiologic studies

**DOI:** 10.1186/1742-5573-2-10

**Published:** 2005-11-25

**Authors:** Betsy Foxman, Lixin Zhang, James S Koopman, Shannon D Manning, Carl F Marrs

**Affiliations:** 1 Department of Epidemiology and Center for Molecular and Clinical Epidemiology of Infectious Diseases, University of Michigan School of Public Health, Ann Arbor, Michigan, USA; 2 National Food Safety/ & Toxicology Center, Michigan State University, East Lansing, Michigan, USA

**Keywords:** molecular epidemiology, methods, bacteria, typing

## Abstract

A wide variety of bacterial typing systems are currently in use that vary greatly with respect to the effort required, cost, reliability and ability to discriminate between bacterial strains. No one technique is optimal for all forms of investigation. We discuss the desired level of discrimination and need for a biologic basis for grouping strains of apparently different types when using bacterial typing techniques for different epidemiologic applications: 1) confirming epidemiologic linkage in outbreak investigations, 2) generating hypotheses about epidemiologic relationships between bacterial strains in the absence of epidemiologic information, and 3) describing the distributions of bacterial types and identifying determinants of those distributions. Inferences made from molecular epidemiologic studies of bacteria depend upon both the typing technique selected and the study design used; thus, choice of typing technique is pivotal for increasing our understanding of the pathogenesis and transmission, and eventual disease prevention.

## Introduction

Ever since Koch discovered how to grow bacteria in pure culture, the laboratory has been an integral component of epidemiologic studies of bacterial diseases. Over time, our ability to discriminate among bacterial strains from the same species has increased, enhancing outbreak investigations and surveillance, studies of the natural history of infection, and our understanding of the transmission, pathogenesis and phylogeny of bacteria.

## Analysis

### Bacterial typing systems

Traditional typing systems for discriminating between bacteria from a single species have been based on phenotype, such as serotype, biotype, phage typing, or antibiogram (susceptibility to one or more antibiotics). More recently, techniques have been developed based on indirect measures of genetic sequence (such as pulsed-field gel electrophoresis (PFGE)) and direct measures of genetic sequence (such as multilocus sequence typing (MLST)). Sequencing an entire bacterial genome, and, using microarray technologies, comparing strains to a reference strain (comparative genomic hybridization) is now technically feasible; however, the cost and time required limits the applicability for most epidemiologic studies. For example, in 2005, total genomic sequencing costs roughly 100 to 500 times more per strain than comparative hybridization (~$100,000 to $500,000 versus ~$1000 to $2000), and MLST (~$140) is quite costly compared to PFGE (~$20). Further, we have yet to characterize the range of variability among bacterial strains of a single species by various techniques, and thus lack an appropriate context for interpreting the observed variation.

Understanding the strengths and weaknesses of the chosen bacterial typing technique enhances interpretation and generalization of study results. A summary of common typing techniques and the relative discriminatory power, repeatability (same test result, given random error, for same analysis on same sample in the same laboratory), reproducibility (same test result, given random error, for same analysis on same sample in a different laboratory), timing and cost is presented in Table 1; techniques have been recently reviewed elsewhere [1-3]. We have ordered techniques from those with the highest to lowest discriminatory power, that is, ability to distribute strains into the greatest number of groups. Thus, if the entire genome of a bacteria is sequenced we will be able to detect even very small differences between strains, for example, changes in gene sequence that do not cause changes in the expressed proteins, such as point mutations that naturally occur over time as the bacteria divides. Common typing techniques used in epidemiologic studies sequence one or more genetic regions, for example multi-locus sequence typing (MLST), or use enzymes to cut part or all of the genome into pieces, for example, pulsed-field gel electrophoresis. The number and size of the pieces correspond to the number and location of restriction sites cut by the enzymes, and thus are an indirect measure of sequence. Other common techniques use the polymerase chain reaction targeted to specific sequences, for example ERIC-PCR; the resulting reactions yield fragments of different sizes, which can be used to discriminate between bacterial types. Generally speaking, sequence-based methods are most repeatable and reproducible. Gel-based methods are less so, because of the inherent variability of the technique [2,3].

**Table 1:** Comparison of Common Bacterial Typing Techniques by Relative Discriminatory Power, Reproducibility, Repeatability, and Whether They Give Information on Dispersed or Focal Parts of the Genome, Time Required and Cost

Typing Technique	Relative discriminatory power	Relative repeatability	Relative reproducibility	Dispersed or focal parts of the genome*	Days required post culture	Relative Cost**	Notes
Sequencing of entire genome	High	High	High	Entire genome	Months to years	Very high	
Comparative hybridization against array containing entire gene sequence	High	Medium to high	Medium to high	Dispersed	Weeks to months	High	Microarrays are increasingly available for human pathogens – not all genes will be present in the sequenced strain
Direct sequencing of one or more genetic regions	Moderate to high (depends on gene choice)	High	High	Focal if only one region	2–3	Equipment: Medium to High Labor & Supplies: Medium to High	Initial selection of target genes might be time consuming.
Multilocus sequence typing (MLST)	Moderate to high (depends on gene choice)	High	High	Dispersed	3+	Equipment: Medium to High Labor & Supplies: High	Initial selection of target genes might be time consuming. Species specific.
Binary typing (presence/absence of selected genes or alleles across the genome)	Moderate to high (depends on gene choice)	High	Potentially High	Dispersed (if chose different genes across the genome)	2–3	Equipment: medium Labor & Supplies: Medium	Reliability dependent on DNA yield and purity
Pulsed-field gel electrophoresis (PFGE)	Moderate to high (depends on number of bands observed)	Medium=> High (depending on species)	Medium =>High	Dispersed	3	Equipment: High Labor & Supplies: High	Discrimination depends on type and number of enzymes selected.
Restriction fragment length polymorphism (RFLP)	Moderate to High (depends on number of bands observed)	Medium=>High	Medium	Dispersed	1–3	Medium	
Amplification of a single target gene specific to a pathogen	Moderate to high (depends on gene choice)	High	Medium=>High	Focal	<1	Equipment: Low to Medium Labor & Supplies: Low	
Amplified fragment length polymorphism (AFLP)	Moderate to high	High	Medium=>High	Dispersed	2	Equipment: Low to Medium Labor & Supplies: Low	
Automated ribotyping	Moderate	High	High	Focal	1	Equipment: High Labor & Supplies: High	Works for most bacterial species
Ribosomal RNA gel electrophoresis	Moderate	High	High	Focal	1	Equipment: Low Labor & Supplies: Medium	
Targeting known repetitive gene sequences (enterobacterial repetitive intergenic consensus sequences (ERIC), repetitive extragenic palindromic sequences (REP), DRE (double repetitive element), BOX, insertional sequence (IS), polymorphic GC-rich repetitive sequences (PGRS))	Low to moderate	Medium	Low	Generally dispersed	1	Equipment: Low to Medium Labor & Supplies: Low	Patterns vary with equipment used
Random primers (randomly amplified polymorphic DNA (RAPD), arbitrary primed PCR (AP-PCR))	Low to moderate	Low	Low	Dispersed	1	Equipment: Low to Medium Labor & Supplies: Low	Patterns vary with equipment used
Restriction endonuclease on a single amplified product	Low to moderate (depends on amplicon)	High	High	Focal	1–2	Equipment: Low to Medium Labor & Supplies: Low	
Plasmid profiles	Low	High	Medium	Focal	1	Equipment: Low Labor & Supplies: Low	

*Focal corresponds to interrogating a single loci. Dispersed means multiple loci are interrogated.

**Per isolate costs in US dollars in 2005, assuming all equipment are available, and the investigator has access to automatic sequencing, for PCR reactions are ~$5, PFGE~$20, MLST ~$140, comparative hybridization~$1000 to $2000 and total genomic sequencing (assuming a strain has already been sequenced)~$100,000 to $500,000.

Note: For a summary and details of these techniques, and assessments of repeatability and reproducibility, see Tenover, 1997 [1], Gurtler and Mayall 2001 [2] and VanBelkum, 2003 [3]. In general, sequence-based methods are most repeatable and reproducible. Gel-based methods are less so, because of the inherent variability of the technique.

Our intention is not to focus on a particular technique, as the techniques continue to change rapidly. Instead, we discuss the strengths and weaknesses of current bacterial typing techniques for particular epidemiologic applications, and provide some insight into what characteristics a typing technique should have when applied to a specific research question. We recognize that choice of a molecular tool is often up to laboratory personnel and not the epidemiologist; however, laboratorians are not always involved in study design or the interpretation of study results (although this is highly desirable). A laboratorian, whose expertise is in a particular typing technique, cannot be expected to give appropriate advice if s/he does not understand the research question asked. Similarly, an epidemiologist cannot appropriately analyze and interpret results of a typing technique if s/he does not understand what it is measuring. Furthermore, if there is a mismatch between typing technique and research question, the study results are less likely to answer the research question. Unfortunately, epidemiologists and laboratorians often have little training in each other's fields, do not share a common vocabulary, and have very different research perspectives. Thus, our goal is to provide guidance for the epidemiologist about working collaboratively with laboratories to choose the appropriate bacterial typing technique, and for interpreting the results.

### Epidemiologic Applications of Bacterial Typing Techniques

Discriminatory power is the average probability that a typing system will assign the same strain type to strains randomly sampled from the same group. In a typical analysis, epidemiologists use questionnaire data to discriminate between groups. For example, if investigating a foodborne outbreak associated with a picnic, then the variable 'ate food at the picnic' will be a poor discriminator of disease risk (as probably all ate), but 'ate potato salad' or even 'ate potatoes' might accurately classify individuals into high and low risk groups (if an ingredient in the potato salad, such as the eggs or mayonnaise, was the culprit). If we classify individuals into groups by all variables measured simultaneously (e.g., age, gender, food preferences, medical history, etc.), then our measure will be highly discriminatory (as each individual might fall into a separate group) – although not necessarily informative with respect to disease risk. Thus, the most discriminatory grouping is not necessarily the most informative, particularly if the groupings are not associated with the outcome of interest.

Bacterial typing techniques are analogous, but may or may not provide an appropriately discriminatory grouping (similar to 'ate potato salad'). We have identified three purposes where molecular typing techniques are applied in epidemiologic studies (Table 2). We give an example of a research goal that relates to each purpose, provide an assessment of the required discriminatory power and need to infer genetic relationships and/or population structure for that particular application. Each purpose is discussed, in turn, below.

**Table 2:** Required Discriminatory Power and Need to Infer Genetic Relationships and/or Population Structure for Various Epidemiologic Applications of Bacterial Typing Techniques

Purpose	Example Research Goal	Discriminatory Power Needed	Need to infer genetic relationships and/or population structure
Confirm epidemiologic linkage	a. Determine if epidemiologically related cases share the identical organism. Result: either support or refute epidemiologic data.	Low	Low
Generate hypotheses about epidemiologic relationships between bacterial strains in the absence of epidemiologic data	a. Determine if time-space clustering surveillance isolates have identical or related genetic types. Result: trigger further epidemiologic investigation of related isolates. b. Determine if outbreak is propagated. Result: trigger investigation into how is spread and/or control actions to stop spread. c. Relate clinical outcomes to strain types or to the presence of transferable genetic material, e.g., antimicrobial resistance on a plasmid. Result: improve patient care.	Moderate to High	Moderate
Describe distribution of bacterial types and identify the determinants of that distribution	a. Test the hypothesis of clonal spread versus independent origin of a particular strain over disparate geographic areas. Result: Better predict emergence and spread of disease. b. Determine flow of infection from one group to another. Result: Public health intervention c. Identification of pathogenic factors. Result: Develop new interventions or therapies specific to those factors	Moderate to High	High

First, however, we wish to point out that bacterial typing is not always the correct classification tool, as outbreaks are not always caused by a single, virulent clone. Contamination of the water or food supply by sewage can lead to an outbreak of diarrhea caused by a variety of different agents [4-6] although clonal outbreaks also occur following sewage contamination [7]. Other examples are the breakdown of abattoir procedures that lead to contamination from cows colonized with diverse agents, or of nursery hygiene procedures allowing transmission from visitors to children.

Further, strain typing results must be interpreted in the context of epidemiologic evidence as well as the characteristics of the bacteria. Neither laboratory nor epidemiologic evidence is definitive, but each validates the other. When epidemiologic evidence suggests contamination arising from diverse sources, stricter molecular typing criteria should *not *be used to classify cases as epidemic related. If typing data suggests a high degree of similarity, epidemiologic evidence should be sought relevant to a single contamination episode.

#### Confirm Epidemiologic Linkage

One of the most common applications of bacterial typing in an epidemiologic study is in the context of an outbreak investigation. Bacterial typing is used to confirm or refute epidemiologic evidence that cases are linked or that a particular food item, water source, or fomite was the source of infection. In this situation the laboratory data is essentially confirmatory and the required discriminatory power and need to infer genetic relationships or structure is low. If there is strong epidemiologic evidence linking a specific food item with disease (common or point source), for example, we often make public health decisions based on that evidence alone – even if there is no supporting laboratory evidence. In the vast majority of foodborne outbreaks, the suspected food is not available for culture and a definitive linkage cannot be demonstrated [8]. Nonetheless, these investigations often successfully identify correctable breaks in hygiene practice. However, even modestly discriminatory techniques are useful since the laboratory evidence confirms the epidemiologic findings. For this type of confirmation, using a rapid and inexpensive technique (like ERIC-PCR) might be preferred since the cost and time associated with a more definitive technique (like MLST) would add little to our understanding of the source of infection or the ultimate policy decision.

#### Generate hypotheses about epidemiologic relationships between bacterial strains in the absence of epidemiologic data

Molecular typing has increased the power of surveillance data to detect outbreaks. The Foodborne Diseases Active Surveillance Network (FoodNet) conducted by the Centers for Disease Control and Prevention uses pulsed-field gel electrophoresis to type surveillance isolates for several foodborne pathogens, including *E. coli *O157:H7, nontyphoidal *Salmonella *serotypes, *Listeria monocytogenes *and *Shigella *[9]. Bacterial typing of space-time clusters has identified unsuspected linkages triggering investigations, as well as demonstrating that apparent clusters were not related, ruling out need for investigation [10].

Molecular typing also facilitates the detection of chains of transmission. Molecular typing led to a reassessment of the epidemiology of tuberculosis in the United States by establishing that tuberculosis does not require prolonged contact but can be transmitted in casual settings [11]. Typing also allows us to relate clinical outcome to strain types, distinguishing recent tuberculosis infection from reactivation of disease, [12] and establishing that an individual can be infected with a second, different tuberculosis strain following initial infection [13].

When the investigator needs to identify potential outbreaks by typing surveillance isolates, or to distinguish between point source and propagated outbreaks, a more discriminatory technique is required. In a common or point source outbreak we expect the causative agent to be similar in all infected persons. Therefore, a more discriminatory technique is necessary to determine if a space-time cluster of isolates detected via surveillance represents a potential outbreak compared to a technique for typing isolates already epidemiologically linked. In a propagated outbreak or when tracking chains of transmission, the genetic sequence of the bacteria may be slightly different at the end compared to the beginning of the outbreak (how fast this occurs depends on the bacteria, however). If the bacteria are naturally competent, i.e., easily uptake DNA from other members of the species, such as non-typeable *Haemophilus influenzae *[14], a highly discriminatory typing technique may erroneously misclassify epidemic cases identified at the end of the epidemic as non-epidemic, particularly if there are no endemic strains available for comparison. Using a typing technique that allows classification consistent with phylogenetic relationships (e.g., MLST), or, if the bacteria is highly recombinant, with clonal complexes, is helpful as there is a biologically meaningful way to group strains (that is, logically collapse groups of related strains). Unfortunately, many typing techniques are analogous to nominal scales, e.g., ERIC: the groups are different from each other, but we cannot say which of the identified groups are more similar than others. Even for PFGE, which can be used to assess relatedness, similarity may vary by choice or number of restriction enzymes used. Further, the published criteria for PFGE relatedness (based on number of matching bands) were intended solely for outbreak situations and when isolates were collected over a short time period (<1 year) and there is an implied epidemiologic linkage [15].

#### Describe distribution of bacterial types and identify the determinants of that distribution

Advances in molecular genetics have facilitated the description of the genetic diversity of bacterial populations. Molecular genetic techniques have been used to distinguish if there have been independent spontaneous mutations leading to antibiotic resistance or if resistance was transmitted between strains via a mobile genetic element. In other applications molecular genetic techniques have determined the flow of infection from one group to another. These descriptive molecular epidemiologic studies often use strains collected from disparate areas and the epidemiologic and clinical information is minimal or non-contributory. In this case the chosen bacterial typing technique must be interpretable in terms of genetic distance (phylogeny) for the given time period and organism. Further, the technique should reflect whether the hypothesis is of clonal spread of a strain or of a mobile genetic element, (e.g., plasmid).

Some typing techniques are based on conserved genes within the bacterial genome, e.g., genes associated with metabolism or other 'housekeeping' functions, and others on more variable genes, e.g., genes associated with virulence. On average, when bacterial strains are compared using a genetic typing technique, there are fewer genetic differences between bacterial strains in the conserved genes than variable genes. Thus, typing techniques based on differences in conserved genes, such as MLST, will place strains into fewer, larger, groups, than typing techniques based on more variable genes, such as PFGE. Put another way, PFGE is generally more discriminatory than MLST.

For bacterial characteristics that are dependent both on the conserved and variable portions of the genome, such as virulence, the use of multiple typing techniques may be helpful, see, for example, [16]. Selection of the appropriate typing technique and a valid interpretation of the results for studies of distribution of bacterial types and the determinants of that distribution is easiest when at least some preliminary data are available. For example, knowledge of the rarity of the observed groups in the community, propensity of the species to acquire insertion elements or phage, the timing of strain collection and the evolutionary clock of the organism, that is, how quickly mutations occur or horizontal elements are acquired provides important information for both technique selection and interpretation of resulting findings.

The identification of pathogenic factors is an exercise in identifying what is different between strains causing and not causing disease. This identification proceeds in the manner of a case-control study with the bacterial agent as the unit of analysis [see, for example, [17]]. Standard epidemiologic study design issues apply: the study population must include both disease-causing and commensal isolates. Most disease-causing strains will predominate in a culture; non-pathogenic, or commensal organisms are often comprised of a mixture of strains of the same species. The investigator must select isolates for study accordingly. For example, *E. coli *is a common bowel inhabitant and is also the most common cause of urinary tract infection. Typically an individual has several *E. coli *strains in the bowel flora but urinary tract infection among outpatients is almost always caused by a single strain. The investigator must decide if the predominant isolate in the bowel flora is the one of interest or if several isolates should be selected for testing. If the objective were to link the bowel to the urinary tract flora, then choosing only the predominant bowel strain would not be sufficient. Identifying common elements generating pathogenicity may be the study objective: when the typing technique is unable to discriminate between pathogenic and diverse commensal isolates, epidemiologic and clinical information should be used to make that distinction, such as grouping together *E. coli *that cause urinary tract infection.

Pathogenicity determinants are often present on transferable genetic material, such as plasmids, pathogenicity islands, phages, etc. Transferable genetic material has a genetic history distinct from the rest of the host bacterial genome. In this case, phylogenetic analyses of these elements can provide useful information. For example, pathogenicity islands (PAIs) have been associated with a variety of conditions, including diarrhea and urinary tract infection [18-20]; specific virulence factor genes found on the PAIs encode for proteins that contribute directly to disease.

## Conclusion

The application and interpretation of bacterial typing tools in epidemiologic studies requires understanding of both the strengths and limitations of the chosen bacterial typing technique as well as the epidemiologic study design to answer the research question. Beyond standard reliability, validity and cost considerations, key characteristics of a typing technique are 1) the ability to discriminate between strains and 2) a biologic basis for grouping strains with apparently different types. The level of discrimination required and need to be able to group strains depends on the research question. Similar to the desirability of including a statistician in the design phase so that the study design will result in appropriate data for the desired analysis, integrating an expert in the different typing techniques during the design phase will improve how well the research protocol fits the question(s) of interest.

## Competing interests

The author(s) declare that they have no competing interests.

## Authors' contributions

BF took the lead on drafting the manuscript, LZ took the lead on Table 1, and JSK outlined Table 2. All authors contributed to discussions leading to the manuscript, critiqued multiple drafts and approved the final manuscript.
